# The relationship between nature connectedness and happiness: a meta-analysis

**DOI:** 10.3389/fpsyg.2014.00976

**Published:** 2014-09-08

**Authors:** Colin A. Capaldi, Raelyne L. Dopko, John M. Zelenski

**Affiliations:** Department of Psychology, Carleton UniversityOttawa, ON, Canada

**Keywords:** nature relatedness, connectedness to nature, happiness, subjective well-being, biophilia, hedonic well-being, meta-analysis, human-nature relationship

## Abstract

Research suggests that contact with nature can be beneficial, for example leading to improvements in mood, cognition, and health. A distinct but related idea is the personality construct of subjective nature connectedness, a stable individual difference in cognitive, affective, and experiential connection with the natural environment. Subjective nature connectedness is a strong predictor of pro-environmental attitudes and behaviors that may also be positively associated with subjective well-being. This meta-analysis was conducted to examine the relationship between nature connectedness and happiness. Based on 30 samples (*n* = 8523), a fixed-effect meta-analysis found a small but significant effect size (*r* = 0.19). Those who are more connected to nature tended to experience more positive affect, vitality, and life satisfaction compared to those less connected to nature. Publication status, year, average age, and percentage of females in the sample were not significant moderators. Vitality had the strongest relationship with nature connectedness (*r* = 0.24), followed by positive affect (*r* = 0.22) and life satisfaction (*r* = 0.17). In terms of specific nature connectedness measures, associations were the strongest between happiness and inclusion of nature in self (*r* = 0.27), compared to nature relatedness (*r* = 0.18) and connectedness to nature (*r* = 0.18). This research highlights the importance of considering personality when examining the psychological benefits of nature. The results suggest that closer human-nature relationships do not have to come at the expense of happiness. Rather, this meta-analysis shows that being connected to nature and feeling happy are, in fact, connected.

## Introduction

Wilson (1984) posits that humans have an inborn tendency to focus on and affiliate with other living things. Termed the *biophilia hypothesis* by Kellert and Wilson (1993), this attraction to life and lifelike processes can be understood through an evolutionary perspective. Because humans have spent almost all of our evolutionary history in the natural environment and have only migrated to urban living in relatively recent times, this attraction, identification, and need to connect to nature is thought to remain in our modern psychology (Kellert and Wilson, 1993). More specifically, it would have been evolutionarily adaptive for our ancestors to be connected to nature in order to survive and thrive in their immediate environmental circumstances. The everyday behaviors of our ancestors such as successfully finding suitable food, water, and shelter, effectively monitoring time and one's spatial location, and avoiding and reacting to predators all heavily relied on paying attention to cues in nature. Thus, individuals who were more connected to the natural world would have had a significant evolutionary advantage over those who were not as connected. To be clear, not all aspects of nature are beneficial and life supporting. For example, Ulrich (1993) reviews instances of *biophobia*, or a biological preparedness to acquire fear of persistently threatening things such as snakes and spiders. Nonetheless, he argues that evidence of biophobia simultaneously suggests the viability of evolved positive responses to the natural world. Evolutionary psychology more generally suggests that modern environments are not optimally suited to minds that evolved in different (more natural) environments (e.g., Barkow et al., 1992; Buss, 2000). Thus, the specific *biophilia* hypothesis is not needed to retain the more general evolutionary idea of modern gaps in optimal human-environment fit.

The gap in nature exposure between our early evolutionary environments and modern life is clear, and appears to be growing. For instance, children are spending less time playing in natural environments compared to previous generations (Clements, 2004; Louv, 2005; England Marketing, 2009) and, in general, individuals from developed nations are spending almost all of their time indoors (Evans and McCoy, 1998; MacKerron and Mourato, 2013). On a broader scale, for the first time in human history, more of the world's population now lives in urban instead of rural areas (United Nations Population Division, 2002). This physical disconnection from the environments in which we evolved in may be having a detrimental impact on our emotional well-being as exposure to nature is associated with increased happiness (Berman et al., 2008, 2012; Mayer et al., 2009; Nisbet and Zelenski, 2011; MacKerron and Mourato, 2013; White et al., 2013).

Beyond these trends, individuals vary along a continuum in their subjective connection to nature (e.g., Mayer and Frantz, 2004). This individual difference, which will be referred to as nature connectedness, can be thought of as trait-like in that it is relatively stable across time and situations (Nisbet et al., 2009). Nevertheless, one's subjective connection to nature can fluctuate (e.g., compare taking a walk outside in nature vs. indoors through tunnels) and be measured at the state level as well (Schultz, 2002; Nisbet and Zelenski, 2011). For the purposes of this paper, nature connectedness will be primarily conceptualized as a trait-like between-person difference.

Consistent personality, attitudinal, behavioral, and well-being differences are found between those who strongly identify with and feel connected to the natural world compared to those who do not. Individuals higher in nature connectedness tend to be more conscientious, extraverted, agreeable, and open (Nisbet et al., 2009; Tam, 2013a). Beyond personality traits, a greater connection to nature is also associated with more pro-environmental attitudes, a greater willingness to engage in sustainable actions, and increased concern about the negative impact of human behavior on the environment (Mayer and Frantz, 2004; Leary et al., 2008; Nisbet et al., 2009; Tam, 2013a). Behaviorally, individuals higher in nature connectedness are more likely to spend time outdoors in nature and engage in a variety of pro-environmental behaviors (e.g., buy “green” products; Mayer and Frantz, 2004; Nisbet et al., 2009; Tam, 2013a). Most relevant to this article, nature connectedness has also been correlated with emotional and psychological well-being (e.g., Nisbet and Zelenski, 2013). The purpose of the current research was to examine the relationship between nature connectedness and happiness in particular by conducting a meta-analysis. The meta-analysis was completed by using correlations to examine the strength of the relationship but not necessarily if one variable causes the other.

An evolutionary history where it was apparently advantageous for our ancestors to be connected to nature and present day variability in nature connectedness appear to be contradictory ideas at first glance, but multiple explanations exist for how both can co-exist. First, similar to how variability in other personality traits can be understood as being the result of cost and benefit trade-offs for fitness (Nettle, 2006), so too can nature connectedness. For example, although conscientiousness is often thought of as a desirable and beneficial personality trait (e.g., it is positively associated with longevity; Friedman et al., 1995), there are certain circumstances where being high in conscientious would be evolutionarily disadvantageous (e.g., missing out on unexpected short-term opportunities; Nettle, 2006). Relatedly, there may have been ways in which being high in nature connectedness was not evolutionarily advantageous (e.g., refusing to kill/eat an animal for sustenance or being too comfortable and not having a reasonable amount of fear of a dangerous predator).

Taking another perspective, although we might have an innate predisposition to connect and identify with the natural world, it may be shaped by early childhood experiences and culture. Orr (1993) raised the idea that there may be a critical period during development where one must have positive experiences in nature in order to develop biophilic beliefs, feelings, and tendencies. In addition, Kellert (1997) believed that biophilia could also be shaped by culture and experiences despite it being inborn. Supporting this, individuals who are higher in nature connectedness as adults recall spending more time in nature during their childhood compared to those who are not as connected to nature (Tam, 2013a). In addition, researchers have found that some groups (e.g., Menominee Native Americans) are more likely to view humans as a part of nature and feel psychologically closer to nature compared to other groups (e.g., European Americans), even at relatively early stages in development (e.g., Bang et al., 2007; Unsworth et al., 2012). This research illustrates that developmental experiences and cultural context can have an influence on our evolved tendency to connect with nature. In sum, the biophilia hypothesis and individual differences in nature connectedness are not contradictory and can logically co-exist to examine and explain the human-nature relationship.

A variety of concepts and measures have been developed in order to assess the human-nature relationship, including commitment to nature (Davis et al., 2009), connectedness to nature (Mayer and Frantz, 2004), connectivity with nature (Dutcher et al., 2007), emotional affinity toward nature (Kals et al., 1999), environmental identity (Clayton, 2003), inclusion of nature in self (Schultz, 2001), and nature relatedness (Nisbet et al., 2009). Through the lens of interdependence theory (Rusbult and Arriaga, 2000), Davis et al. (2009) defined commitment to nature as a “psychological attachment to and long-term orientation toward the natural world” (p. 174) and adapted the commitment scale by Rusbult et al. (1998) which originally assessed commitment to a close partner. Mayer and Frantz (2004) described connectedness to nature as a “measure of an individuals' trait levels of feeling emotionally connected to the natural world” (p. 503) and is explicitly conceptualized as assessing the affective component of the human-nature connection. Another clearly affective nature connectedness construct is emotional affinity toward nature, which was developed by Kals et al. (1999) and involves pleasant feelings of inclination toward nature such as oneness and love. Inclusion of nature in self was developed by Schultz (2001) who adapted the Inclusion of Other in Self scale (Aron et al., 1992) in order “to measure the extent to which an individual includes nature within his or her cognitive representative of self” (Schultz and Tabanico, 2007, p. 1221). With one of its items being the Inclusion of Nature in Self scale, connectivity with nature is defined by Dutcher et al. (2007) as “a sense of sameness between the self, others, and nature” (p. 474). The multidimensional construct of environmental identity, which Clayton (2003) likens to other collective identities that people have, is conceptualized as a feeling of connection to the natural environment and the belief that the environment is an important part of one's self-concept. Lastly, nature relatedness is another multidimensional construct that involves one's “affective, cognitive, and physical relationship with the natural world” (Nisbet et al., 2009, p. 719).

Despite these different concepts and measures, they all appear to be assessing slightly different expressions of the same underlying construct (i.e., one's subjective connection to nature). To support this, they are all highly correlated with one another and associated with other personality characteristics, measures of well-being, and environmental attitudes and behaviors in a relatively similar manner (see Tam, 2013a). For these reasons, no distinctions will be made between these concepts in this paper and nature connectedness will be used as an umbrella term for all of them.

A common line of research for many in this area is the investigation of the relationship between nature connectedness and well-being (e.g., Mayer and Frantz, 2004; Howell et al., 2011; Nisbet and Zelenski, 2011). Well-being and the path to its attainment have traditionally and typically been conceptualized in one of two ways by philosophers and psychologists (Grinde, 2012). From a hedonic perspective, well-being consists of the pleasantness of an individual's experiences and is achieved through the maximization of pleasure and the satisfaction of desires (Kahneman, 1999; Fredrickson, 2001). Subjective well-being, another term for happiness in the hedonic approach, consists of an affective component (i.e., the presence of positive emotional experiences and the absence of negative ones) and a cognitive component (i.e., the evaluation of one's life as satisfying; Diener and Lucas, 1999; Diener, 2009). Specific measures used to assess hedonic well-being include the Positive and Negative Affect Schedule (Watson et al., 1988), the Subjective Happiness Scale (Lyubomirsky and Lepper, 1999), and the Satisfaction with Life Scale (Diener et al., 1985). In contrast, from a eudaimonic perspective, well-being is more about following one's deeply held values and realizing one's fullest potential (Waterman, 1993; Ryff, 1995). As an example, psychological well-being is a construct that is thought to constitute eudaimonic well-being and consists of six facets of actualization including mastery, life purpose, autonomy, self-acceptance, positive relatedness, and personal growth (Ryff and Keyes, 1995). Despite the contentious history between these two perspectives, hedonic and eudaimonic well-being indicators tend to be positively correlated and can influence one another implying that they are not mutually exclusive but overlapping and distinct (King et al., 2006; Waterman, 2008; Huta and Ryan, 2010). Furthermore, individuals high in hedonic and eudaimonic motives tend to experience the greatest amount of overall well-being and are considered to be flourishing (Huta and Ryan, 2010; Forgeard et al., 2011). Nonetheless, due to its more targeted definition, established assessment tools, and common usage compared to the eudaimonic approach (Kashdan et al., 2008), this meta-analysis primarily focused on hedonic measures of well-being.

Although events can influence an individual's present mood state, most have only a limited long-term impact on one's happiness (Steel et al., 2008, but see Diener et al., 2006 for exceptions). In fact, subjective well-being tends to be relatively stable over time (Diener and Lucas, 1999; Lyubomirsky et al., 2005; Nes et al., 2006). Relatedly, subjective well-being is associated with particular personality traits. Similar to nature connectedness, subjective well-being is consistently positively associated with extraversion, agreeableness, and conscientiousness, but unlike nature connectedness it is also negatively correlated with neuroticism (Steel et al., 2008). Lastly, subjective well-being can predict important life outcomes such as health, longevity, and disease (Williams and Schneiderman, 2002; Lyubomirsky et al., 2005; Chida and Steptoe, 2008).

There are several reasons why one would expect nature connectedness to be positively associated with subjective well-being. First, being and feeling connected in general consistently predicts well-being (Ryan and Deci, 2001). For instance, consider social connectedness. A rich and fulfilling social life is a commonality found in the lives of very happy people (Diener and Seligman, 2002). Relatedly, those who are higher in the personality traits of extraversion and agreeableness tend to experience more positive emotions compared to those who are lower in these characteristics (Steel et al., 2008). Within individuals, daily fluctuations in feelings of social relatedness predict changes in subjective well-being (Reis et al., 2000). In contrast, loneliness and shyness are negatively correlated with happiness (Booth et al., 1992) and social exclusion has been found to activate similar brain regions as physical pain (Eisenberg et al., 2003). These findings have led some to argue that social connectedness is a prerequisite for happiness and a basic human need (Baumeister and Leary, 1995). Having a connection with nature may function similarly and also promote well-being. It is important to note that there appears to be something else beyond mere general subjective connectedness which explains nature connectedness' relationship with happiness. When one controls for other connections (e.g., family or culture), nature connectedness still significantly predicts happiness (Zelenski and Nisbet, 2014).

Additionally, individuals who are higher in nature connectedness may seek out more opportunities to reap the psychological benefits associated with nature exposure, or, from a biophilia perspective, satisfy the need to affiliate with other living things. In support of this, nature connectedness is positively associated with nature contact (e.g., frequency of time spent outdoors and in nature) and interaction with other living things (e.g., pet ownership; Nisbet et al., 2009), and there is a substantial amount of evidence that shows that exposure to nature leads to increased happiness (Berman et al., 2008, 2012; Mayer et al., 2009; Nisbet and Zelenski, 2011; White et al., 2013).

There are also plausible reasons to expect an effect in the opposite direction. As previously mentioned, nature connectedness consistently predicts pro-environmental attitudes and concern about the environment (Mayer and Frantz, 2004; Leary et al., 2008; Nisbet et al., 2009; Tam, 2013a). Individuals who incorporate nature into their sense of self may view harm done to nature as harm done to themselves (Mayer and Frantz, 2004). As knowledge, awareness, certainty, and salience concerning the negative impacts that climate change will have on the environment and life on Earth increase, being more connected to nature could conceivably hamper happiness instead of promoting it (Doherty and Clayton, 2011). In fact, a quarter of Americans feel depressed or guilty about the issue of global warming and those who are most alarmed about climate change are more likely to feel afraid, angry, sad, and disgusted (Maibach et al., 2009). The term *eco-anxiety* has even been used by some in the media to reflect the worry and concern about global warming that some individuals have, along with self-reported symptoms of sleeplessness, loss of appetite, weakness, irritability, and panic attacks (Nobel, 2007). Furthermore, models of grieving and mourning following a loss have been applied to individuals' reaction to learning about and accepting global warming and making changes in lifestyle to minimize one's carbon footprint (Randall, 2009). Given the greater environmental concern that seems to accompany a subjective connection to nature, negative emotions and distress may be more frequently experienced by those higher in nature connectedness. From this perspective, one might predict that nature connectedness might be negatively associated with happiness. It is also possible that there is no relationship between one's subjective connection to nature and subjective well-being (e.g., because the positive and negative processes cancel each other, on average).

Although a previous meta-analysis has been published that examined whether exposure to natural environments has a positive impact on health and well-being (Bowler et al., 2010), no meta-analyses have been conducted that have comprehensively investigated whether the trait of nature connectedness is associated with happiness. The purpose of this study was to test whether the relationship between these two constructs was significant, to provide an estimate of its effect, and to determine whether there was significant variability across samples. This is necessary as the association between measures of nature connectedness and happiness appear to vary considerably, with correlation coefficients ranging from −0.01 (Nisbet et al., 2011) to 0.42 (Zelenski and Nisbet, 2014) in the published research literature. We hypothesized that there would be a small but significant relationship between nature connectedness and happiness. This was a realistic estimate as it tends to be the average or median effect size in many areas of psychology (Sarason et al., 1975; Lipsey and Wilson, 1993; Richard et al., 2003).

In addition, moderator analyses were conducted on publication status, year, age, gender, type of happiness, and measure of nature connectedness. Publication status was analyzed as a moderator in order to determine if there is a publication bias in this area of research (i.e., published studies reporting a stronger relationship between nature connectedness and happiness compared to unpublished ones). Year was analyzed as a moderator because of the recent attention given to the decline effect—the observation that many scientific findings diminish with time (Schooler, 2011). Age and gender were analyzed as moderators because being older and being female tend to be associated with higher environmental concern, attitudes, and behaviors (e.g., Grønhøj and Thøgersen, 2009; Scannell and Gifford, 2013). In order to examine whether the different measures of well-being and nature connectedness accounted for any of the variability across samples, separate meta-analyses were run for the most common types of happiness (i.e., positive affect, life satisfaction, and vitality) and nature connectedness (i.e., connectedness to nature, inclusion of nature in self, and nature relatedness).

## Methods

### Inclusion and exclusion criteria

To be included in this meta-analysis, studies had to employ at least one measure of nature connectedness and at least one measure of happiness, and report on their relationship. Only explicit self-report trait measures of identification with nature were included (see Table 1). In contrast, implicit or state measures of nature connectedness were excluded in order to minimize commensurability (i.e., grouping substantially different measures together; Sharpe, 1997; Cortina, 2003). For instance, the average correlation between explicit self-report measures and the implicit association task is 0.24, which is substantially lower than the correlations found between explicit measures of nature connectedness (Hofmann et al., 2005; Tam, 2013a). In addition, state nature connectedness was excluded because a previous meta-analysis has already examined the impacts of nature exposure on emotional functioning (Bowler et al., 2010). Studies that artificially dichotomized nature connectedness were excluded as well to avoid all the problems that are associated with the dichotomization of quantitative variables (see MacCallum et al., 2002).

**Table 1:** **Nature connectedness measures included in meta-analysis**.

**Measure**	**Citation**	**Sample number**
Allo-inclusive identity	Leary et al., 2008	4.2, 5.1, 6
Commitment to nature	Davis et al., 2009	18.1
Connectedness to nature	Mayer and Frantz, 2004	3.1, 3.2, 3.5, 4.1, 4.2, 5.1, 5.2, 7.4, 15, 18.1, 19, 20.1, 20.2
Connectedness with nature—single item	Cervinka et al., 2012	3.1, 3.2, 3.5
Connectivity to nature	Dutcher et al., 2007	18.1
Emotional affinity toward nature	Kals et al., 1999	18.1
Environmental identity	Clayton, 2003	18.1
Inclusion of nature in self	Schultz, 2001	9, 16, 17.4, 18.1, 21.1a, 21.1b
Nature relatedness	Nisbet et al., 2009	1, 2a, 2b, 4.2, 5.1, 8, 9, 10, 11, 12, 13, 14.1, 14.2, 18.1, 21.1a, 21.1b, 21.2

See Table 3 for studies associated with each sample number.

Regarding the second construct of interest, studies that employed either explicit self-report state or trait measures of subjective well-being were included in the meta-analysis (see Table 2). Studies that measured eudaimonic well-being (e.g., self-acceptance), hedonistic values, or implicit measures of happiness were excluded in order to reduce commensurability and maintain a targeted focus on hedonic well-being. Nevertheless, vitality, which is defined as the positive feeling of being alive, alert, and energetic (Ryan and Frederick, 1997), was included in the current meta-analysis. Although it is theoretically conceptualized as a eudaimonic construct and is associated with other measures of eudaimonic well-being (e.g., self-actualization; Ryan and Frederick, 1997), subjective vitality is predicted by both hedonic and eudaimonic motives (Huta and Ryan, 2010), as well as hedonic behaviors (but not eudaimonic ones; Henderson et al., 2013). Furthermore, there appears to be similar conceptual overlap between vitality and some of the other high arousal positive emotions included in measures such as the Positive Affect and Negative Affect Scale (e.g., excited, enthusiastic, alert, attentive, and active; Watson et al., 1988). For these reasons, measures of vitality were included in this meta-analysis. As the focus of this meta-analysis is on positive emotional functioning, measures of negative affect were excluded. Studies that artificially dichotomized happiness were excluded as well due to the dichotomization problems that were previously alluded to.

**Table 2:** **Happiness measures included in meta-analysis**.

**Measure**	**Citation**	**Sample number**
Affect-adjective scale	Diener and Emmons, 1985	1, 2b
Calm, contentment, and peacefulness—single item	Nisbet, 2013a,b	11, 12
Current mood scale from the multidimensional comfort questionnaire	Steyer et al., 1997	3.1
Emotional well-being	Keyes, 2005	4.1, 4.2, 5.1, 5.2
Happy—single item	Ajzen and Driver, 1992	17.4
Nature positive affects	Nisbet, 2011	9, 10, 21.1a, 21.1b, 21.2
Percent happy	Fordyce, 1988	9
Positive and negative affect schedule	Watson et al., 1988	8, 9, 10, 13, 14.1, 14.2, 15, 19, 21.1a, 21.1b, 21.2
Satisfaction with life scale	Diener et al., 1985	1, 2a, 2b, 3.2, 6, 7.4, 9, 13, 14.1, 14.2, 16, 18.1, 19, 21.1a, 21.1b, 21.2
Scale of positive and negative experience	Diener et al., 2010	20.1, 20.2
Steen happiness index	Seligman et al., 2005	5.1, 5.2
Subjective happiness scale	Lyubomirsky and Lepper, 1999	9, 13, 18.1, 21.1a, 21.1b, 21.2
Subjective vitality scale	Ryan and Frederick, 1997	8, 9, 10, 13, 20.1, 20.2, 21.1a, 21.1b, 21.2
Vital, energetic, and enthusiastic—single item	Nisbet, 2013a,b	11, 12
Vitality scale from the short form (36) health survey	Bullinger and Kirchberger, 1989	3.5, 15

See Table 3 for studies associated with each sample number.

All age groups were included as eligible samples because there was no theoretical or practical reason to exclude any in particular. For this same reason, no exclusions were made based on the country where the study was conducted, the language it was written in, or the time when it was conducted. Relatedly, the study had to provide sufficient information to code an effect size and its variance (i.e., correlation coefficient and sample size) to be included. Qualitative studies were excluded and samples sizes had to be above 10 to be included. Lastly, experimental designs were included only if they provided a baseline measure of the relationship between connectedness to nature and happiness prior to any experimental manipulations.

### Search strategies

Numerous methods were used to identify studies. Abstracts were searched in the *PsycINFO* and *Dissertation and Theses Full Text* electronic databases using the various names given for nature connectedness as the search terms: commitment to nature, connectedness to nature, connectivity with nature, emotional affinity toward nature, environmental identity, inclusion of nature in self, and nature relatedness. Reference lists of studies that met the inclusion/exclusion criteria were investigated, as well as the studies that cited them. Authors who conducted studies that measured nature connectedness and happiness but did not report on their relationship were contacted to obtain the necessary statistical information. Requests for additional findings were sent out in May 2013 using the email listserv for Division 34 of the American Psychological Association and in June 2013 using the Conservation Psychology email listserv.

### Coding procedure

A standard coding form and explicit rules outlined in a coding manual that was developed for the current meta-analysis were used for each sample (see Supplementary Material). The standard coding forms contained a cover sheet that was completed for each non-overlapping sample, along with a basic study descriptives form and a sample information form. Specific effect sizes were coded on individual effect size forms. If a sample had multiple measures of nature connectedness and/or happiness, a weighted average of the effect sizes was calculated for that sample. In total, 140 effect sizes were coded from the 30 unique samples with each sample having its own overall effect size. These were used in subsequent analyses in order to ensure that the independence of observations principle was maintained.

Interrater reliability analyses were conducted on all of the non-overlapping samples by the first and second authors. The first author developed the coding manual and coded all of the studies, and then trained the second author as the secondary coder. Minor clarifications and updates were made to the coding manual after the two coders compared their coding of the first couple of studies. The raters coded all of the studies separately and then had multiple meetings where disagreements were identified and consensus ratings were reached.

The two raters coded 124 common effect sizes with high levels of agreement (absolute intraclass correlation [ICC] based on single rater = 0.99). Eleven effect sizes were coded by the first author but not the second and an additional five effect sizes were coded by the second author but not the first. Out of the 140 effect sizes coded by the raters, a consensus was reached that 127 of them should be included in the meta-analysis. High levels of agreement were also found for the other continuous variables that were coded (i.e., year, sample size, percentage of females, and average age of sample), with ICC values ranging from 0.98 to 1.0. When possible, Cohen's Kappa was computed for the categorical variables (*n* = 28) and were found to range from 0.21 to 1 (*M* = 0.91). Following conventions outlined in Landis and Koch (1977), the strength of agreement was almost perfect (i.e., above 0.80) for the vast majority of the categorical variables (*n* = 24) and substantial (i.e., between 0.60 and 0.80) for all the rest of them excluding one. The coding of the happiness measure as state, trait, or mixed had the uniquely low interrater reliability of 0.21. Nonetheless, the overall percent agreement for the coding of this variable was 87.90% and the majority of disagreements occurred early on in the coding process before clarifications were made to the coding manual or due to a rater forgetting to code this variable and leaving it blank. In general, the interrater reliability was relatively high which supports the notion that other raters who followed the same coding manual would code the samples in a consistent manner and end up with similar results.

### Statistical methods

#### Effect size

Because the relationship between two continuous variables was being examined, correlation coefficients were the effect size used to summarize the relationship between nature connectedness and happiness in this meta-analysis. Because some of the correlation coefficients were expected to be above 0.30, correlation coefficients were transformed into Fisher's Z values before being meta-analyzed. This transformation ensured that the variance of the effect size would be solely based on the sample size and not the magnitude of the effect size as well (Borenstein et al., 2009). For ease of interpretation, all the results involving Fisher's Z values have been retransformed into correlation coefficients. Following the conventions outlined in Cohen (1988), correlation coefficients of 0.10 were considered small, 0.30 were considered moderate, and 0.50 were considered large.

#### Aggregation of findings

Both fixed-effect and random-effects meta-analyses were conducted (Borenstein et al., 2009). The fixed-effect model assumes that there is one true effect size and that variability across samples is sampling error. The random-effects model assumes that there is no one true effect size, but a distribution of effect sizes, and variability across samples is real and not just sampling error. The random-effects model allows one to generalize beyond the samples included in the meta-analysis, while the fixed-effect model does not. Despite its advantages, the random-effects model is a more conservative test and unstable when the number of samples is smaller than 30 (Overton, 1998; Schulze, 2007). Both fixed-effect and random-effects meta-analyses were conducted in order to account for the advantages and disadvantages of each and increase confidence in consistent results. Regardless of the model, the effect size of each sample was weighted by the inverse of its variance.

Cochran's *Q* statistic was computed to determine whether there was significantly more variability across samples than what one would expect by chance (Borenstein et al., 2009). To determine the percentage of variability across samples that is beyond what one would expect by chance, *I*^2^ was obtained. Following recommendations outlined in Higgins et al. (2003), an *I*^2^ value of 25% was considered low variability, 50% was considered moderate, and 75% was considered high.

Because outliers can distort the results of a meta-analysis, extreme effect sizes were identified by following the rules developed in Hanson and Bussière (1998). First, the effect size must be either the highest or lowest in magnitude. Second, Cochran's *Q* statistic must be significant. Third, the effect size must account for more than half of *Q*. Fourth, there must be more than three samples. If a potential outlier was found, the results with and without that sample were reported but interpretations were based on the latter. A sample with an extremely large sample size relative to the rest of the samples can also have a large impact on the results of a meta-analysis. Following the rule used in other meta-analyses (e.g., Helmus et al., 2013), the weight of the largest sample was reduced to be only 50% larger than the weight of the second largest sample if the variability across samples was found to be significant.

#### Moderator analyses

For categorical moderators, fixed-effect between-level *Q* moderator analyses were conducted (Borenstein et al., 2009). The between-level *Q* was obtained to determine whether the moderator significantly accounted for the unexplained variability across samples. Fixed-effect was chosen because moderator analyses tend to have low power and fixed-effect moderator analyses provide more power than random-effects. In addition, the *Q* statistic is easier to interpret in fixed-effect models. For continuous moderators, fixed-effect meta-regression was conducted (Borenstein et al., 2009). Meta-regression was conducted to determine whether the moderator is a significant predictor of effect size. Fixed-effect assumes that the moderators completely explain the effect size of the samples and that there is no residual heterogeneity. It was chosen over random-effects because of its higher power.

## Results

### Overview of included studies

As of August 2nd, 2013, 30 non-overlapping samples from 21 studies were identified. Descriptive information for each of the included samples can be found in Table 3. The total sample size was 8523. The sample sizes ranged from 22 to 2224 (*M* = 284.10, *SD* = 384.47, *Mdn* = 215). Many of the samples came from Canada (46.7%), followed by the United States (20%), Europe (10%), Asia (3.3%), and mixed locations (10%). All the studies were written in English. The samples ranged in average age from 19.48 years to 63.42 years (*M* = 31.91, *SD* = 11.37). The percentage of females in each sample ranged from 38.62 to 86.10% (*M* = 65.33, *SD* = 11.84). University/college students made up 33.3% of the samples, while 40% of samples were community members and 6.7% of samples contained a mix. The years of the studies ranged from 2004 to 2014. Samples were coded as published if they came from a journal article or book chapter. Using this criterion, 60% of the samples were published and 40% were unpublished. Samples were coded as peer reviewed if they came from a dissertation/thesis or journal article. Using this criterion, 73.3% of the samples were peer-reviewed and 26.7% were not.

**Table 3:** **Descriptive information for included samples**.

**Sample number**	**Study**	***N***	**Location**	**Mean age (years)**	**% Female**	**Published**
1	Aitken and Pelletier, 2013a	272	Canada	–	–	No
2a	Aitken and Pelletier, 2013b	189	Canada	–	–	No
2b	Aitken and Pelletier, 2013b	369	Canada	–	–	No
3.1	Cervinka et al., 2012	94	Europe	37.30	57.40	Yes
3.2	Cervinka et al., 2012	119	Europe	36.00	52.10	Yes
3.5	Cervinka et al., 2012	101	Europe	34.30	54.50	Yes
4.1	Howell et al., 2011	437	Canada	22.17	69.40	Yes
4.2	Howell et al., 2011	262	Canada	20.39	68.00	Yes
5.1	Howell et al., 2013	311	Canada	22.07	68.00	Yes
5.2	Howell et al., 2013	227	Canada	23.29	63.00	Yes
6	Leary et al., 2008	148	–	–	–	Yes
7.4	Mayer and Frantz, 2004	135	USA	36.00	65.93	Yes
8	Nisbet, 2005	354	Canada	20.03	59.90	No
9	Nisbet, 2011	207	Mixed	27.81	77.80	No
10	Nisbet, Unpublished data	22	–	–	–	No
11	Nisbet, 2013a	2,225	Canada	45.76	83.80	No
12	Nisbet, 2013b	341	Canada	46.79	86.10	No
13	Nisbet, Unpublished data	110	–	–	–	No
14.1	Nisbet et al., 2011	184	Canada	19.48	67.40	Yes
14.2	Nisbet et al., 2011	145	Canada	42.37	38.62	Yes
15	Okvat, 2011	50	USA	63.42	84.00	No
16	Reist, 2004	357	Mixed	36.42	66.00	No
17.4	Schultz and Tabanico, 2007	39	USA	–	67.50	Yes
18.1	Tam, 2013a	322	Asia	20.36	45.34	Yes
19	Trull, 2008	66	Canada	–	56.06	No
20.1	Wolsko and Lindberg, 2013	265	USA	30.11	62.90	Yes
20.2	Wolsko and Lindberg, 2013	223	USA	33.30	61.40	Yes
21.1a	Zelenski and Nisbet, 2014	331	Canada	20.50	73.10	Yes
21.1b	Zelenski and Nisbet, 2014	415	Mixed	32.20	79.70	Yes
21.2	Zelenski and Nisbet, 2014	204	USA	–	60.00	Yes

*Samples were given numbers based on their order in the reference list. If there were multiple studies within the same paper, the numbers to the right of the decimal indicate which specific study the sample came from. Letters indicate that there were multiple samples within the same study. For example, study number 21.1b indicates that it was the second sample within the first study of the twenty-first paper. The sample size for each sample is the one associated with its overall/averaged effect size*.

### Overall effect size and statistical significance

**Figure 1:**
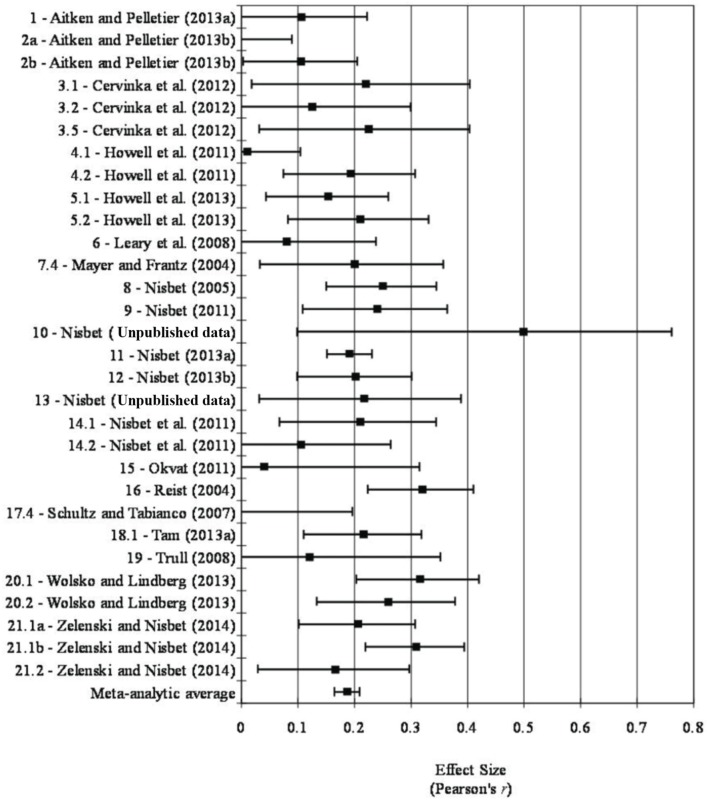
**Forest plot**.

Figure 1 is a forest plot which shows the effect size and confidence interval associated with each sample and the meta-analytic average from the fixed-effect meta-analysis. Table 4 shows the results of both the fixed-effect and random-effects meta-analysis. As one can see, both models produced relatively consistent results. More specifically, a small mean weighted effect size was found between nature connectedness and happiness in the fixed-effect [*r* = 0.19, 95% CI (0.16, 0.21), *k* = 30, *n* = 8523] and random-effects models [*r* = 0.18, 95% CI (0.15, 0.22), *k* = 30, *n* = 8523]. Because both of the 95% confidence intervals did not include zero, one can conclude that this small mean weighted effect size was significant at the *p* < 0.05 level.

**Table 4:** **Meta-analysis results**.

**Fixed-effect**			**Random-effects**						
	**95% CI**			**95% CI**					
***r***	***LL***	***UL***	***r***	***LL***	***UL***	***Q***	***I*^2^ (%)**	***k***	***n***
0.19	0.16	0.21	0.18	0.15	0.22	64.29^***^	54.89	30	8523

*** *p < 0.001*.

### Analysis of heterogeneity

The variability across samples was significant (*Q* = 64.29, *df* = 29, *p* < 0.001) and the *I*^2^ indicated that 54.89% of the observed variability was beyond what would be expected by chance. In other words, it would be reasonable to conclude that there was a moderate amount of variability across samples. This implies that there may be some variables moderating the magnitude of the effect size.

### Outliers and extremely large samples

Following the rules developed in Hanson and Bussière (1998), no outliers were identified. Although Cochran's *Q* was significant and there were more than three samples, the samples with the highest and lowest effect sizes did not account for more than half of the *Q* statistic. When the sample with the lowest effect size (Schultz and Tabanico, 2007; *r* = −0.13) was removed from the meta-analysis, the *Q* statistic did not decrease by 50% (*Q* = 60.76). Relatedly, when the sample with the highest effect size (Nisbet, Unpublished data; *r* = 0.50) was removed from the meta-analysis, the *Q* statistic did not decrease by 50% (*Q* = 61.83). For these reasons, all the samples identified were included in the overall meta-analysis.

Nisbet (2013a) can be considered an extremely large sample as it contributed over a quarter of the total participants in this meta-analysis and its sample weight was more than five times the size of the second largest weight (2221 vs. 434). Following the rules outlined in the methods section, the sample weight of Nisbet (2013a) was artificially reduced to be only 50% larger than the second largest sample weight (i.e., 651). This is what was used in the overall meta-analysis and the moderator analyses.

### Investigation of potential moderators

Moderator analyses were conducted to examine whether publication status, gender, year, age, type of happiness, and measure of nature connectedness accounted for the significant variability across samples.

#### Publication status

To examine whether there was a publication bias, fixed-effect between-level *Q* moderator analyses were run with publication status (i.e., published vs. unpublished) as the categorical moderator. Table 5 shows the results of this moderator analysis. Because the between-level *Q* statistic is distributed as a chi-square with the degrees of freedom being the number of levels of the categorical variable minus one, the critical value for this moderator analysis is 3.84 for *p* < 0.05 at a degrees of freedom of 1. As the between-level *Q* did not exceed the critical value (between-level *Q* = 0.01, *df* = 1, *p* = 0.92), one can conclude that publication bias is probably not an issue for this research topic.

**Table 5:** **Fixed-effect between-level *Q* moderator analysis for publication bias**.

	***r***	**95% CI**	***Q***	***I*^2^ (%)**	***k***	***n***	**Samples Included**
Overall	0.19	[0.16, 0.21]	64.29^***^	54.89	30	8523	All
Unpublished	0.19	[0.15, 0.22]	28.84^**^	61.85	12	4561	1, 2a, 2b, 8, 9, 10, 11, 12, 13, 15, 16, 19
Published	0.19	[0.16, 0.22]	35.44^**^	52.03	18	3962	3.1, 3.2, 3.5, 4.1, 4.2, 5.1, 5.2, 6, 7.4, 14.1, 14.2, 17.4, 18.1, 20.1, 20.2, 21.1a, 21.1b, 21.2
*Q*_between_			0.01				

** p < 0.01;

*** *p < 0.001*.

#### Gender

In order to investigate whether gender moderates the relationship between nature connectedness and happiness, a fixed-effect meta-regression was conducted with percentage of females in the sample as the predictor variable. Percentage of females in the sample was not a significant predictor of effect size (slope = 0.0004, *SE* = 0.00113, *Z* = 0.35, *p* = 0.73, *k* = 24, *n* = 7413).

#### Year

In order to investigate whether the relationship between nature connectedness and happiness is influenced by the year, fixed-effect meta-regressions were conducted with year as the predictor variable. Year was not a significant predictor of effect size (slope = −0.00479, *SE* = 0.00412, *Z* = −1.16, *p* = 0.25, *k* = 30, *n* = 8523). Thus, one can conclude that the decline effect is probably not an issue for this research topic.

#### Age

In order to examine whether the relationship between nature connectedness and happiness stays the same throughout the lifespan, fixed-effect meta-regressions were conducted with average age of the sample as the predictor variable. Average age was not a significant predictor of effect size (slope = 0.00064, *SE* = 0.00134, *Z* = 0.48, *p* = 0.63, *k* = 21, *n* = 7104).

#### Type of happiness

The relationship between nature connectedness and happiness may depend on how happiness is defined and measured. Because some of the samples used multiple measures of happiness, conducting moderator analyses on this variable would violate the principle of independence. For this reason, general patterns were observed by conducting separate meta-analyses for the three main types of happiness in this study: positive affect, life satisfaction, and vitality. Samples that did not contain a particular type of happiness measure were excluded from that respective meta-analysis and overall effect sizes for each type were calculated for the remaining samples. Both fixed-effect and random-effects meta-analyses were conducted.

***Positive affect***. A small mean weighted effect size was found between nature connectedness and positive affect in the fixed-effect [*r* = 0.22, 95% CI (0.19, 0.25), *k* = 19, *n* = 5926] and random-effects models [*r* = 0.22, 95% CI (0.17, 0.26), *k* = 19, *n* = 5926]. The variability across samples was significant (*Q* = 40.69, *df* = 18, *p* = 0.002) and moderate as the *I*^2^ indicated that 55.77% of the observed variability was beyond what would be expected by chance.

***Life satisfaction***. A small mean weighted effect size was found between nature connectedness and life satisfaction in the fixed-effect [*r* = 0.17, 95% CI (0.14, 0.20), *k* = 16, *n* = 3575] and random-effects models [*r* = 0.16, 95% CI (0.11, 0.20), *k* = 16, *n* = 3575]. The variability across samples was significant (*Q* = 32.17, *df* = 15, *p* = 0.006) and moderate as the *I*^2^ indicated that 53.37% of the observed variability was beyond what would be expected by chance.

***Vitality***. A small mean weighted effect size was found between nature connectedness and vitality in the fixed-effect [*r* = 0.24, 95% CI (0.21, 0.27), *k* = 13, *n* = 4824] and random-effects models [*r* = 0.24, 95% CI (0.19, 0.29), *k* = 13, *n* = 4824]. The variability across samples was significant (*Q* = 23.77, *df* = 12, *p* = 0.02) and moderate as the *I*^2^ indicated that 49.51% of the observed variability was beyond what would be expected by chance.

#### Measure of nature connectedness

The relationship between nature connectedness and happiness may depend on the measure used to assess one's connection to nature. Because some of the samples used multiple measures of nature connectedness, conducting moderator analyses on this variable would violate the principle of independence. For this reason, general patterns were observed by conducting separate meta-analyses for the three most commonly used measures of nature connectedness: the connectedness to nature scale (Mayer and Frantz, 2004), the inclusion of nature in self-scale (Schultz, 2001), and the nature relatedness scale (Nisbet et al., 2009). Samples that did not contain a particular type of nature connectedness measure were excluded from that respective meta-analysis and overall effect sizes for each were calculated for the remaining samples. Both fixed-effect and random-effects meta-analyses were conducted.

***Connectedness to nature***. A small mean weighted effect size was found between happiness and connectedness to nature in the fixed-effect [*r* = 0.18, 95% CI (0.14, 0.22), *k* = 13, *n* = 2615] and random-effects models (*r* = 0.18, 95% CI (0.13, 0.24), *k* = 13, *n* = 2615]. The variability across samples was significant (*Q* = 23.80, *df* = 12, *p* = 0.02) and moderate as the *I*^2^ indicated that 49.59% of the observed variability was beyond what would be expected by chance.

***Inclusion of nature in self***. A small mean weighted effect size was found between happiness and inclusion of nature in self in the fixed-effect [*r* = 0.27, 95% CI (0.23, 0.32), *k* = 6, *n* = 1671] and random-effects models [*r* = 0.25, 95% CI (0.15, 0.35), *k* = 6, *n* = 1671]. The variability across samples was significant (*Q* = 21.59, *df* = 5, *p* < 0.001) and high as the *I*^2^ indicated that 76.84% of the observed variability was beyond what would be expected by chance.

***Nature relatedness***. A small mean weighted effect size was found between happiness and nature relatedness in the fixed-effect [*r* = 0.18, 95% CI (0.16, 0.21), *k* = 17, *n* = 6255] and random-effects models [*r* = 0.18, 95% CI (0.14, 0.22), *k* = 17, *n* = 6255]. The variability across samples was significant (*Q* = 28.63, *df* = 16, *p* = 0.03) and moderate as the *I*^2^ indicated that 44.12% of the observed variability was beyond what would be expected by chance.

## Discussion

The purpose of this study was to provide a quantitative summary of the literature on the link between nature connectedness and happiness. Auspiciously, a fairly clear picture emerged. The relationship between nature connectedness and happiness appears to be positive and significant. In general, individuals who are more connected to nature tend to be happier.

Demographic characteristics, such as gender and age, did not moderate this relationship despite previous research finding that being older and female tends to be associated with increased pro-environmental concern, attitudes, and behaviors (e.g., Grønhøj and Thøgersen, 2009; Scannell and Gifford, 2013). It appears that possible age or gender differences in nature connectedness or well-being did not impact the association between the two. Publication bias did not appear to be an issue, nor was any evidence for the decline effect found—thus increasing confidence in the current meta-analytic summary effect.

How happiness was defined and measured did appear to have an influence on the magnitude of the effect size, with vitality being the most strongly associated with nature connectedness, followed by positive affect and life satisfaction. Nature's restorative effects might explain why vitality has the strongest relationship with nature connectedness (Kaplan, 1995). Beyond its ability to improve emotional functioning, exposure to natural environments has also been shown to alleviate cognitive fatigue, improve attention, and increase feelings of vitality (Berman et al., 2008; Ryan et al., 2010; Nisbet et al., 2011). As those who are higher in nature connectedness are more likely to spend time in nature, they may be beneficiaries of both the affective and revitalizing effects of natural environments, which is reflected by nature connectedness' even stronger association with vitality compared to the other measures of happiness. Vitality being a traditionally eudaimonic measure of well-being might also explain its higher mean weighted effect size. Increased concern for the environment and engagement in sustainable behaviors might carry more hedonic than eudaimonic costs to well-being (Venhoeven et al., 2013) and this may manifest in slightly lower correlations with the more classically hedonic measures of well-being (i.e., positive affect and life satisfaction). The variability in mean weighted effect sizes may be partially due to vitality and positive affect being affective components of well-being, while life satisfaction is more of a cognitive component (Diener and Lucas, 1999; Diener, 2009). Although the different measures of subjective well-being are typically conceived of as assessing the same underlying construct and factor analysis supports this (Sandvik et al., 1993), correlations between different measures of subjective well-being (e.g., recalled positive affect and life satisfaction) tend to be moderate in magnitude (Lucas et al., 1996) indicating that the constructs are not identical (Kim-Prieto et al., 2005). The non-shared variance between measures of subjective well-being might partly explain some of the varying results. Lastly, different proportions of the nature connectedness measures included within each of the meta-analyses could have conceivably influenced or confounded the results. This is unlikely as the percentages of nature connectedness measures within each type of happiness meta-analysis remained fairly consistent, with nature relatedness being the most common (ranging from being in 69.2% of the samples in the vitality meta-analysis to 68.4% of the samples in the positive affect meta-analysis), followed by inclusion of nature in self (ranging from being in 31.3% of the samples in the life satisfaction meta-analysis to 21.1% of the samples in the positive affect meta-analysis), and connectedness to nature (ranging from being in 30.8% of the samples in the vitality meta-analysis to 25% of the samples in the life satisfaction meta-analysis) Regardless of all these explanations, the effect size from each of the meta-analyses examining type of happiness remained relatively similar in magnitude (i.e., small) and all of the fixed-effect confidence intervals either almost overlapped (i.e., vitality and life satisfaction) or did overlap (i.e., vitality and positive affect, as well as positive affect and life satisfaction).

How nature connectedness was defined and measured also appeared to have an influence on the magnitude of the effect size, with inclusion of nature in self-having a particularly stronger relationship with happiness compared to nature relatedness and nature connectedness. This is consistent with the pattern of results found in Zelenski and Nisbet (2014) where zero-order correlations between measures of happiness and nature connectedness were larger for inclusion of nature in self than nature relatedness. One possible explanation for this difference is that inclusion of nature in self may also assess general connectedness more than other measures of nature connectedness which might more precisely tap individuals' subjective connection to nature (Zelenski and Nisbet, 2014). Considering the aforementioned well-being benefits associated with social connection (Ryan and Deci, 2001), more overlap between the general construct of connectedness and inclusion of nature in self could increase the latter's relationship with happiness. In fact, inclusion of nature in self, compared to nature relatedness, has been found to correlate substantially more with general connectedness (Zelenski and Nisbet, 2014). In contrast to these patterns of results, Tam (2013a) found that inclusion of nature in self consistently shared the weakest association with subjective well-being out of all the nature connectedness measures. As Tam (2013a) was the one study on this topic that was conducted in Asia, cross-cultural differences may account for these inconsistencies. Related to this point, researchers in this area should attempt to recruit participants from more diverse backgrounds beyond western, educated, industrialized, rich, and democratic societies (Henrich et al., 2010), as the majority of samples in this meta-analysis came from Canada and the USA. This is especially pertinent given the cultural differences that have been observed in how people conceptualize the relationship between humans and nature (e.g., Bang et al., 2007; Unsworth et al., 2012). The differential distribution of happiness measures is an unlikely explanation for the varying effect sizes found in the separate nature connectedness meta-analyses as the majority of overall/averaged effect sizes within each were based on mixed measures of positive affect, vitality, and/or life satisfaction. Regardless of these explanations, the confidence intervals either almost overlapped (i.e., in the fixed-effect meta-analyses) or did overlap (i.e., in the random-effects meta-analyses). It should also be noted that the number of samples was fairly low (*k* = 6) and the variability between samples was high in the inclusion of nature in self meta-analysis.

Although the overall effect size from this meta-analysis can be considered small when one follows conventions (Cohen, 1988), as was first noted by Mayer and Frantz (2004), it is similar in size to other variables widely thought to have a positive relationship with happiness, such as personal income within countries (Haring et al., 1984; Diener et al., 1993), education (Witter et al., 1984; Diener et al., 1993), religiosity (Witter et al., 1985; Hackney and Sanders, 2003; Diener et al., 2011), marital status (Haring-Hidore et al., 1985; Diener et al., 2000), volunteering (Thoits and Hewitt, 2001), and physical attractiveness (Diener et al., 1995; Plaut et al., 2009). Furthermore, it is similar in magnitude to the association between subjective well-being and some personality traits such as conscientiousness and agreeableness (DeNeve and Cooper, 1998; Steel et al., 2008). More generally, the overall effect size between nature connectedness and happiness is similar to the average result found in social psychology (i.e., *r* = 0.21; Richard et al., 2003). Thus, a person's connection to nature should be considered an important construct when discussing happiness and vice versa.

It should be noted that correlation does not equal causation. Higher nature connectedness may cause increased happiness, higher happiness may cause increased nature connectedness, or a third variable might be leading to changes in both variables. Studies have been conducted that employ experimental designs and attempt to manipulate nature connectedness and/or happiness (e.g., Nisbet, 2011). Using statistical mediation analyses, some studies have found that exposure to nature increases nature connectedness because it promotes positive affect (Nisbet and Zelenski, 2011), while other studies have found that nature exposure increases people's emotional well-being partially due to increased nature connectedness (Mayer et al., 2009). Due to the problems associated with meditation analyses (see Bullock et al., 2010) and the fact that these studies confound nature exposure and positive emotions, future research is needed to determine the directionality of this relationship. To our knowledge, no studies have experimentally manipulated happiness (without nature) to examine whether it would lead to a greater sense of connection to the natural world, above and beyond other subjective connections (cf. Zelenski and Nisbet, 2014). This could offer a valuable extension to Fredrickson's (2004) broaden-and-build theory of positive emotions beyond social bonds to connections with nature as well.

Strong subjective connections to nature may begin in childhood. However, the association between childhood experiences and an individual's level of nature connectedness as an adult has only been established through recall in self-reports (Tam, 2013a) thus far. Conducting longitudinal studies that follow individuals across the lifespan would allow researchers to more accurately answer whether childhood contact with nature predicts nature connectedness years later. This could test Orr's (1993) idea of a critical period for developing biophilia and could help explain individual differences in people's subjective connection to nature. The relationship that nature connectedness has with negative emotional functioning, physical health, and cognitive abilities are also promising areas of investigation (cf. Bowler et al., 2010).

Although vitality was included in the operational definition of happiness in this paper, an examination of the relationship between nature connectedness and other constructs that are commonly thought of as eudaimonic well-being such as autonomy, personal growth, self-acceptance, purpose in life, environmental mastery, and positive relations (Ryff, 1989), would provide a fruitful avenue for future research and meta-analysis in and of itself. Of the fewer studies that have looked at this relationship, they tend to find a positive association between nature connectedness and measures of eudaimonic well-being as well (Howell et al., 2011, 2013; Nisbet et al., 2011; Zelenski and Nisbet, 2014). It would be interesting to examine whether this relationship differs significantly in strength from the association found between nature connectedness and hedonic well-being. A review of how pro-environmental behaviors can influence well-being in both positive and negatives ways by Venhoeven et al. (2013) hints that it might as research “suggests that engaging in pro-environmental behavior may have especially negative consequences for hedonic well-being, but mainly positive consequences for eudaimonic well-being” (p. 1380). Although there are circumstances where this may not hold true, the eudaimonic motive of “doing something good” like engaging in pro-environmental behaviors, even when it is difficult, costly, or time-consuming, logically may lead to eudaimonic but not hedonic well-being. As nature connectedness predicts sustainable attitudes and behaviors (Mayer and Frantz, 2004; Leary et al., 2008; Nisbet et al., 2009; Tam, 2013a), this suggests that the relationship between nature connectedness and eudaimonic well-being may be even stronger. That nature connectedness was most strongly associated with vitality also seems to offer preliminary support for this prediction.

Nevertheless, this meta-analysis provides results that run somewhat counter to what one would predict based on Venhoeven's (2013) review as subjective connection to nature is associated with greater hedonic well-being, not less. This suggests that although some aspects of the human-nature relationship have the potential to detract from our happiness (e.g., some pro-environmental behaviors), other aspects may compensate and result in a net increase (e.g., a subjective connection to and contact with nature). Instead of potentially difficult, time-consuming, and costly pro-environmental behaviors coming at an expense to our subjective well-being, sustainable behaviors might be a pleasant expression of a trait (i.e., nature connectedness) that promotes overall positive emotional functioning. This has important implications as we attempt find solutions to many of the problems we face in the twenty-first century, such as climate change and the rising burden of disease of mental illness (World Health Organization, 2001).

Similar to how all the different conceptualizations of well-being were not included in this meta-analysis, other constructs relating to the human-nature relationship (e.g., dispositional empathy with nature; Tam, 2013b) may have been overlooked that warrant further investigation. Moreover, opportunities to develop novel constructs beyond nature connectedness could be expanded by applying existing psychological theories and concepts about human interpersonal relations to the human-nature domain (Tam, 2014). For instance, attachment theory could be extended to a person's attachment to nature, with different attachment styles (i.e., secure, anxious-preoccupied, dismissive-avoidant, and fearful-avoidant; Bartholomew and Horowitz, 1991) potentially being assessed and used to predict variables like connection to nature, environmental attitudes, and likelihood of engaging in sustainable behaviors.

Despite the unambiguous findings of the current meta-analysis and the preferences for nature that people commonly hold (Frumkin, 2001), research suggests that individuals tend to commit affective forecasting errors and underestimate the hedonic benefits that being in nature will bring them (Nisbet and Zelenski, 2011). Given that people are spending the vast majority of their time indoors (Evans and McCoy, 1998; MacKerron and Mourato, 2013) and the increasing urbanization of the world's population (United Nations Population Division, 2002), many of us may be missing out on the beneficial effects of connecting to nature in the moment and in general. This could be contributing to a decrease in not only our own well-being, but that of our planet as well. The current meta-analysis provides further evidence that a sustainable future and a happy future are compatible and symbiotic, not mutually exclusive.

### Conflict of interest statement

The authors declare that the research was conducted in the absence of any commercial or financial relationships that could be construed as a potential conflict of interest.
