# Supplementary material for: The relationship between nature connectedness and happiness: a meta-analysis
Source: Front Psychol. 2014 Sep 8;5:976. doi: 10.3389/fpsyg.2014.00976 (PMC4157607; doi:10.3389/fpsyg.2014.00976)
Supplement: Supplementary file 1 [file CodingManual.DOCX]

**CODING MANUAL**

**HAPPINESS AND NATURE CONNECTEDNESS META-ANALYSIS**

**(HANCMA)**

**Colin Capaldi**

**2014**

**Last updated January 26^th^, 2014**

For additional information, please contact Colin Capaldi,

[colin_capaldi@carleton.ca](mailto:colin_capaldi@carleton.ca)

**CODING MANUAL**

**HAPPINESS AND NATURE CONNECTEDNESS META-ANALYSIS**

**(HANCMA)**

Inclusion/Exclusion Criteria

For a study to be included in the 2013 meta-analysis on the relationship between nature connectedness and happiness, it must meet the following criteria:

- Studies must employ some measure of nature connectedness.
  - There must be an explicit self-report trait measure of nature connectedness for a study to be included.
    - Examples include the Nature Relatedness Scale, the Connectedness to Nature Scale, and the Inclusion of Nature in Self Scale.
  - Studies that employ implicit measures of nature connectedness will be excluded.
    - An example of an implicit measure is the Implicit Association Task.
  - Studies that employ state measures of nature connectedness will be excluded.
    - An example of a state measure is an item that asks “How connected to nature do you feel *at this moment*?”.
  - Studies that artificially dichotomize nature connectedness will be excluded.
- Studies must employ some measure of happiness.
  - There must be an explicit self-report state or trait measure of hedonic well-being for a study to be included.
    - Examples include the Positive and Negative Affect Schedule, the Subjective Happiness Scale, the Vitality Scale and the Satisfaction with Life Scale.
    - Measures of negative affect will be excluded.
  - Studies that measure eudaimonic well-being will be excluded.
    - Examples of eudaimonic well-being are self-acceptance, autonomy, purpose in life, and environmental mastery.
  - Studies that employ implicit measures of happiness will be excluded.
    - An example of an implicit measure is the Implicit Association Task.
  - Studies that artificially dichotomize happiness will be excluded.
- Studies must report on the relationship between a measure of nature connectedness and measure happiness in order to be included.
- Studies must provide sufficient statistical information to code an effect size and its variance (i.e., correlation coefficient and sample size).
- Studies must include a sample size of at least 10.
- Case studies and qualitative studies will be excluded.
- Studies that employ experimental designs will be included only if they provide a baseline measure of the relationship between connectedness to nature and happiness prior to any experimental manipulations.
- No studies will be excluded based on where it was conducted, what language it was written in, nor the time it was conducted in.
- All age groups will be included.

**COVER SHEET HANCMA 2013**

STUDY NUMBER: _____________________________________________________________

TITLE: _______________________________________________________________________

____________________________________________________________________________________________________________________________________________________________

AUTHOR(S):______________________________________________________________________________________________________________________________________________________________________________________________________________________________

YEAR(S): _____________________________________________________________________

SOURCE: _____________________________________________________________________

______________________________________________________________________________

______________________________________________________________________________

NOTES: ______________________________________________________________________

__________________________________________________________________________________________________________________________________________________________________________________________________________________________________________

# OF SHEETS/EFFECT SIZES CODED: ____________________________________________

DATE: _______________________________________________________________________

CODER: ______________________________________________________________________

**BASIC STUDY DESCRIPTIVES**

- STUDY_NUMBER : ________________________
- PUBLISHED: ________________________

*Was the study published?*

0 = no

1 = yes

- PEER_REVIEW: _____________________

*Was the study peer reviewed?*

0 = no

1 = yes

- ADDITIONAL_INFO: _________________

*Was additional information obtained to code the effect size?*

0 = no

1 = yes

- YEAR: _____________________________

*What was the year in which the study was published, released, or finished?*

- LOCATION: _________________________

*Where was the study conducted?*

0 = Canada

1 = United States

2 = Europe

3 = Asia

4 = Australia

5 = Other

- EXPERIMENTAL: ___________________

*Did the study employ an experimental or quasi-experimental design?*

0 = no

1 = yes

**SAMPLE INFORMATION**

- SAMPLE_SIZE: ______________________

*What was the overall sample size?*

- PERCENT_FEMALE: _________________%

*What percentage of the overall sample size above is female (up to 2 decimal points)?*

- STUDENTS: _________________________

*Were the participants university/college students?*

0 = no

1 = yes

2 = mixed

- AVERAGE_AGE: ____________________

*What was the mean age of participants in years (up to 2 decimal points)*?

- MAJORITY_ETHNICITY: _____________

*What was the most common ethnicity in the sample?*

0 = White

1 = Black

2 = Aboriginal

3 = Asian

4 = Other

**EFFECT SIZE CODING**

- Study #: _____________________________
- Effect size # _____ of _____

*Out of the total number of effect sizes for that study*.

- Is this an overall/averaged effect size for the study? _____

0 = no

1 = yes

- How were the two variables measured? For the overall/averaged effect size, select all that apply below. For specific effect sizes, select one measure for each variable.

| Measure(s) of nature connectedness: | Measure(s) of happiness: |
| --- | --- |
| _____ Nature Relatedness  _____ Connectedness to Nature  _____ Inclusion of Nature in Self  _____ Environmental Identity  _____ Emotional Affinity toward Nature  _____ Connectivity to Nature  _____ Commitment to Nature  Other: ___________________________________  Other: ___________________________________ | _____ Positive and Negative Affect  Schedule    _____ Subjective Happiness Scale  _____ Vitality Scale  _____ Satisfaction with Life Scale  _____ Percent Happy  Other: ___________________________________  Other: ___________________________________  Other: **___________________________________** |

- What is the correlation coefficient? *r* = ____________________

(up to 3 decimal points)

- What is the sample size? *n* = ____________________
- What is the Fisher’s Z value? *Z =* ____________________
  (up to 3 decimal points)
- Page #: _____________________
- Table #: ____________________

Study Information

A cover sheet, basic study descriptives, and sample information will be filled out for each sample. Below are additional rules on how to deal with issues that will likely arise when attempting to code studies:

- Each paper that meets the inclusion/exclusion criteria will be given a paper number. Initially, papers should be given a number based on their order in the reference list. If the paper contains more than one study, then each study within that paper will also receive a study number. As an example, a study labelled ‘3.2’ would indicate that it is the second study from the third paper. If overlapping samples are found, the sample with the largest sample size will be included.
- The year of the study will be coded as the year in which the entire paper was published, released, or completed.
- The source refers to where the study was found. If it is from a journal article, then the journal it was published in is the source. If the study is from a chapter in a book, then the title of the book is the source. If the study was from a thesis or dissertation, then one should simply write “Master’s thesis” or “Doctoral dissertation” as the source. If the study was a paper or poster presented at an academic conference, then one should write “Paper/poster presented at Name of Conference” as the source. If the study was unpublished data obtained from a researcher, then one should write “Unpublished data” as the source.
- Studies will be coded as published if they come from a journal article or a book chapter. In press journal articles will be coded as published. Dissertations/theses, conference presentations, government reports, and manuscripts will not be considered published. If additional information was required to code the effect size (e.g., contacting the authors), then it will not be coded as published.
- Only the studies from dissertations/theses and journal articles will be coded as peer-reviewed. If additional information was required to code the effect size, then it will not be coded as peer-reviewed.
- Samples will be coded as requiring additional information if informal means were used to obtain information about the effect size (e.g., email).
- The year will be coded as the year it was released in-print. If it hasn’t been released in-print yet, the year will be coded as the year it was released online. If it hasn’t been published in-print or online, then the year will be coded as the year the study was conducted. For samples from conference presentations, the year will be coded as the year of the conference.
- If a sample contains participants from multiple countries (e.g., the study was conducted online), the location will be coded as other.
- The overall sample size will be coded as the sample size associated with the overall/average effect size.
- To calculate percent female when the gender of some participants is unknown, the number of known females will be divided by the total sample size (including the number of participants with unknown gender) and then multiplied by 100.
- If a sample contains university/college students and community members, it will be coded as mixed.

All the rest of the coding is expected to be fairly straightforward. Coders will consult the coding forms which contain detailed instructions. Additional rules and solutions to issues will be added to the coding manual if/when they arise.

Effect Size Statistic

Since the relationship between two continuous variables is being investigated, correlation coefficients will be the effect size statistic for this meta-analysis. Because some of the correlation coefficients are expected to be above .30, correlations will be transformed into Fisher’s Z values using an online calculator (<http://onlinestatbook.com/calculators/fisher_z.html>). This transformation will ensure that the variance of the effect size will be solely based on the sample size. Below are the formulas that will be used to transform correlation coefficients into Fisher’s Z values and calculate its standard error and variance.


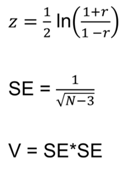


Rules for Effect Size Coding

If a study meets all the inclusion/exclusion criteria above but does not explicitly report on the relationship between a measure of nature connectedness and happiness and/or the sample size, one will attempt to contact and obtain unreported information from the authors of the study. This will be the first approach taken when only qualitative statements are made about the relationship between these two variables (i.e., they just say that there is a significant or non-significant relationship between nature connectedness and happiness). If the authors cannot be contacted or cannot provide specific statistical information, the following rules for extrapolations will be followed:

- If the authors just report that the relationship is non-significant, the effect size will be coded as a zero.
- If the authors report that the relationship is non-significant but indicate a general direction, the midpoint between zero and the minimum value needed for significance will be coded as the effect size.
- If the authors report that the relationship is significant and indicate a general direction, the minimum value needed for significance will be calculated, along with a range of plausible effect sizes based on other studies, and a random value will be selected from that range as an effect size.
- If the authors cannot be contacted or they cannot provide specific statistical information or qualitative statements about the relationship, then the study must be excluded from the meta-analysis.

If a study contains multiple measures of nature connectedness and/or happiness, a weighted average of the effect sizes will be calculated to maintain the independence of observations in the overall meta-analysis. The individual effect sizes for each measure will also be coded for subsequent moderator analyses. The weighted effect size will be calculated using the following general formula (where ES = *r*):

Below are additional rules and clarifications on how to deal with issues that will likely arise when attempting to code effect sizes:

- An effect size will be considered an overall/averaged effect size if a sample only contains one effect size or if the effect is a weighted average of effect sizes in that sample.
- Overall/average effect sizes should be the first effect size coded in each sample.
- When bivariate, partial, and semipartial correlation coefficients are reported in a paper, only the bivariate correlation coefficient will be coded.
- When the relationship between subscales of nature connectedness and happiness are reported, along with the relationship between overall nature connectedness and happiness, the correlation coefficient associated with the overall nature connectedness measure will be coded and the correlation coefficients associated with the subscales will be ignored.
- When an averaged overall effect size is made up of various measures with different sample sizes, the average sample size will be calculated and coded for n (to the nearest whole number).
- When the sample sizes for the measure of nature connectedness and the measure of happiness are not the same, the smaller sample will be coded as n.
- Coders should indicate whether the measure of happiness assessed state or trait happiness. If the effect size is an overall/averaged effect size that is made up of multiple measures of happiness that assess both state and trait happiness, one should indicate that it is mixed. For example, when happiness measures have instructions that include “right now” or “at this moment” it should be coded as state. In contrast, when happiness measures have instructions that include “in general” or “in the past week” it should be coded as trait.
- When page numbers are not present within a paper (e.g., the paper is in press), then the page of the PDF document that contains the information about that effect size will be coded as the page number.
